# Prevalence of internet addiction in tunisian adolescents

**DOI:** 10.1192/j.eurpsy.2021.1527

**Published:** 2021-08-13

**Authors:** M. Daoud, S. Omri, R. Feki, N. Smaoui, L. Zouari, J. Ben Thabet, M. Maalej Bouali, N. Charfi, M. Maalej

**Affiliations:** 1 Department Of Psychiatry “c”, Universty Hospital of Hedi Chaker, sfax, Tunisia; 2 Psychiatry C Department, Hedi chaker University hospital, sfax, Tunisia

**Keywords:** Addiction, Internet, adolescents

## Abstract

**Introduction:**

Internet addiction (IA) is a significant public health issue among adolescents. There is considerable evidence that IA is associated with various psychosocial harms.

**Objectives:**

The aim of this study was to measure the prevalence of internet addiction among secondary school.

**Methods:**

This cross-sectional study was conducted among 152 students enrolled in secondary school. The participants had filled the Internet Addiction Test of Young and a data file regarding the socio-demographic information, physical and information about the internet access and use.

**Results:**

The sample consisted of 90 males and 62 females with a mean age of 13.14 ± 1.2 years. The majority of participants had their own smartphone (83.6%). The average duration of connection among participants was 5.3 hours per day. The prevalence of IA was 14,5%. Results showed that 46,8% feel their internet use significantly hinders their family relationships. Twenty participants (13,2%) reported that they connected to the internet while they were in classroom. The prevalence of IA was higher among boys than girls (p= 0.018). There was, also, a significant relation between IA and having academic difficulties (p=0.037).

**Conclusions:**

The prevalence of IA is elevated in Tunisia. Many negative consequences are identified. Urgent measures should be taken to counter the problem.

